# Accessible AI-powered poultry disease diagnostics: development, validation, and web deployment of a farmer-friendly MobileNet-based system for coccidiosis and salmonella detection in resource-constrained settings

**DOI:** 10.1016/j.psj.2025.106338

**Published:** 2025-12-29

**Authors:** Al Momen Pranta

**Affiliations:** Department of Animal Science, Bangladesh Agricultural University, Mymensingh 2202, Bangladesh

**Keywords:** poultry disease classification, transfer learning, MobileNetV2, Support Vector Machine

## Abstract

Automated detection of diseases in the poultry farming industry is seriously challenged in resource-limited farming environments where computational resources and technical expertise are scarce. This work fills this gap, via systematic evaluation of lightweight transfer learning architectures for practicalpro deployment. Two state-of-the-art pre-trained Convolutional Neural Network (CNN) models, MobileNetV2 and MobileNetV3Small, were tested along with three traditional Machine Learning Models (Support Vector Machine (SVM), Logistic Regression (LR) and K-Nearest Neighbours (KNN)) by using a balanced dataset containing 6436 images of faecal samples from three classes: Coccidiosis, Salmonella and Healthy. MobileNetV2-SVM showed better performance with 96.17% test accuracy (96% precision, recall, and F1-score), which was much better than other pipelines based on MobileNetV3Small (maximum 83.94% accuracy). The optimized pipeline achieves real-time inference at 61 milliseconds per image, enabling deployment on standard hardware. A publicly accessible web-based application was developed, allowing farmers and veterinary practitioners to perform smartphone-based disease classification without specialized expertise, democratizing AI-powered diagnostics for resource- limited agricultural settings. This research establishes a systematic benchmark for lightweight feature extraction architectures combined with traditional machine learning classifiers in poultry disease detection and demonstrates that practical, farmer-accessible AI diagnostics can achieve clinical-grade accuracy even in resource constrained environments.

## Introduction

The poultry industry is under increasing pressure to meet the growing demand for poultry products due to population growth and changing dietary preferences. This explosion in demand, in conjunction with the implementation of Industry 4.0 technologies in agricultural practices, requires effective disease management systems to sustain production. However, intensive farming practices have led to the development of suitable conditions for the rapid transmission of disease which infectious diseases such as Coccidiosis, Salmonellosis and Newcastle disease are causing significant economic losses due to reduced productivity and increased death rates (World Organisation for Animal Health, 2018). The impact is especially extreme in resource-constrained settings where traditional methods of diagnostic practices, including visual inspection and clinical assessment by veterinarians, are heavily relied upon and are labour-intensive and non- scalable to large-scale operations (Msoffe et al., 2018).

Recent developments in the area of deep learning have transformed the field of automated classification of diseases in the poultry farming industry and provided better precision and processing power. Early investigations by Wang et al. (2019) established the basic viable approach of Convolutional Neural Networks (CNNs) for pathogenic detection with Faster R- CNN achieving 93.3% precision in avian pathogenic detection in faecal samples. This fundamental work was further verified and improved by Mbelwa et al. (2021), who reported 94% validation accuracy, providing important benchmarks for future architectural refinements. In parallel efforts, Machuve et al. (2022) used a set of architectures: MobileNetV2 and Xception, achieving impressive test classification accuracies of 98.02% and 98.24% respectively, through well-organized hyperparameter optimization protocols. Contemporary research trends have been increasingly focused on tackling the issue of computational efficiency within the context of high diagnostic accuracy. Liu et al. (2023) pioneered development of the lightweight PoultryNet architecture based on MobileNetV3 with an accuracy of 97.77% while reducing the computational requirements significantly. The efficacy of the transfer learning methodologies has been further demonstrated in a variety of architectural implementations with Bingol et al. (2023) validating MobileNetV2 architecture providing true prediction rates of 97.1%, while Degu et al. (2023) EfficientNet B5 demonstrated 98.7% classification accuracy for diverse pathological conditions.

Despite these major developments, there are still some major challenges in optimizing poultry disease detection frameworks. Existing studies have mainly been concentrated on either heavyweight architecture with high computational requirements or lightweight models with reduced accuracy. Furthermore, the systematic investigation of the feature extraction capabilities of multiple pre-trained CNN architectures using traditional machine learning classifiers is under- explored through the context of poultry disease classification. The identification of the best architectural configurations that can balance diagnostic accuracy with feasibility of deployment in resource-constrained environments is a major research gap. This study addresses these limitations by systematically evaluating two state-of-the-art pre-trained CNN architectures, i.e., MobileNetV2 and MobileNetV3Small, as feature extractors, with 3 classical machine learning classifiers, i.e., Support Vector Machine (SVM), Logistic Regression (LR) and K-Nearest Neighbours (KNN). The proposed methodological pipeline combines transfer learning capabilities of lightweight CNN architectures with traditional classification algorithms which are novel contributions to the existing literature. Specifically, whereas the variants of MobileNet have been investigated separately in previous studies, the comparative analysis of their feature extraction capability when coupled systematically with several different classification algorithms has not been reported so far under poultry disease detection scenarios.

The overall goal of this study is to determine an optimized configuration of automated poultry disease classification that can produce high computational accuracy while ensuring high computational efficiency that can be deployed in poultry operations for practical purposes. By limiting the scope to just three very important classes of disease (Coccidiosis, Salmonellosis, Healthy), this research offers specific solutions to the most common health issues in poultry farming. The use of lightweight mobile architectures guarantees feasibility of edge computing applications, allowing real-time detection of diseases via the use of mobile devices in the field. This study shows that strategic combination of efficient feature extraction with proper use of classifying algorithm can provide performance metrics similar to heavyweight architectures while ensuring practicality for deployment, therefore aiding in development of accessible automated diagnostic systems for poultry health management in resource constrained agricultural systems.

## Materials and methods

### Dataset for poultry diseases

The dataset used for this study was taken from a large poultry disease diagnostics project undertaken in Tanzania's Arusha and Kilimanjaro regions between September 2020 and February 2021, with particular reference to the most common health conditions which affect commercial poultry operations. The dataset encompasses 8,067 total images collected from layers, cross, and indigenous chicken breeds across numerous independent farms in two distinct administrative regions over a 6-month period, ensuring diverse representation of management systems and environmental conditions. Images were captured using mobile devices equipped with the Open Data Kit (ODK) application following standardized protocols established for the original dataset collection (Machuve et al., 2022). According to the dataset documentation, images were captured using ordinary smartphones (including Tecno, Infinix, Huawei, and Samsung models) with ODK application interface for consistent data collection across multiple farms in Arusha and Kilimanjaro regions during September 2020 - February 2021. The ODK framework provided automated standardization of image capture parameters, geo-location tagging, and quality control protocols across all collection sites. While specific technical parameters (exact resolution settings, focal length, and detailed lighting conditions) are not comprehensively documented in the publicly available dataset, the multi-device approach using ODK reflects real-world deployment scenarios where farmers utilize readily available smartphones for disease detection, enhancing the practical relevance of our validation results for resource-constrained agricultural settings.

**Fig. 1:**
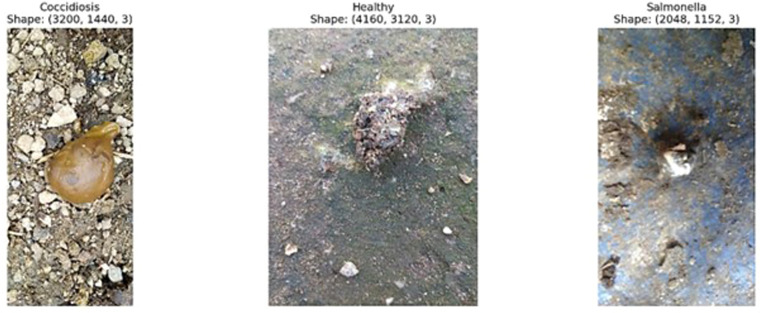
Training dataset samples for disease classification: A) Coccidiosis-infected poultry feces; B) Healthy poultry feces; C) Salmonella-infected poultry feces.

The dataset encompasses three disease classes with established diagnostic protocols (Machuve et al., 2022). Coccidiosis diagnosis was confirmed through standard parasitological examination involving microscopic identification of Eimeria species oocysts in fecal samples with oocyst counting procedures (Johnson and Reid, 1970; Grilli et al., 2018). Salmonella diagnosis was established through controlled experimental inoculation with known bacterial strains followed by fecal sample collection one-week post-inoculation, with additional Polymerase Chain Reaction (PCR) diagnostic procedures employing specific primers for target DNA amplification and confirmation through gel electrophoresis techniques (Oliveira et al., 2003). A subset of 1,255 images received comprehensive laboratory validation through PCR diagnostics at Nelson Mandela African Institution of Science and Technology, while the remaining 6,812 farm-labeled images were annotated by veterinary professionals based on established clinical criteria, with healthy samples representing fecal material confirmed negative for target pathogens serving as the baseline reference class. The dataset covers three different classes of disease: Coccidiosis affected samples ('Coccidiosis'), which represented parasitic infections caused by Eimeria species; Salmonella- induced samples ('Salmonella'), which represented bacterial infections; and normal faecal material from healthy chickens ('Healthy'), which was taken as the base line reference class. Sample images that represent each class are shown in Fig. 1. The first data set contained 6436 images that were distributed among classes with the following numbers: Coccidiosis: 2103 images, Healthy samples: 2057 images, Salmonella samples: 2276 images. The distribution of the dataset is described in Table 1.

**Table 1:** Classification accuracy on validation and test sets.

Pre-trained Model	Classifier	Train_CA (%)	Validation_CA (%)	Test_CA (%)
MobileNetV2	SVM	100	98.03	96.17
MobileNetV2	LR	99.45	96.99	95.23
MobileNetV2	KNN	100	95.75	88.29
MobileNetV3Small	SVM	81.23	78.53	83.94
MobileNetV3Small	LR	73.69	69.40	80.41
MobileNetV3Small	KNN	100	60.48	77.93

Analysis of the first dataset showed a relatively balanced class distribution, and the coefficient of variation of class distribution showed very little imbalance (less than 10% from the mean). This near-equilibrium in class representation minimizes risks of classifier bias on overrepresented classes and therefore helps in achieving unbiased model training without the need of any elaborate augmentation strategies (Johnson and Khoshgoftaar, 2019). However, to improve model robustness and generalization abilities, we employed data augmentation with ImageDataGenerator with judiciously calibrated parameters.

The augmentation strategy involved a number of transformations that were designed to mimic real-world imaging variations while maintaining diagnostic features. Specifically, pixel values were normalized to [0,1] by rescaling (rescale = 1.255), so that the input was standardized when used for neural network processing. Geometrical transformations were random rotations, range +−30 deg (rotationrange=30), horizontal and vertical shifts up to 20% (widthshiftrange=0.2,heightshiftrange=0.2), shear transformations up to 0.2 rad (shearrange=0.2) and zoom variations within +−20% zoomrange (zoomrange=0.2). Additionally, the ability to flip horizontally was enabled (horizontalflip=True) to compensate for arbitrary image orientation during capture of fields. The nearest neighbor interpolation technique was used as a fill mode to preserve the integrity of the pixels during transformations.

Quality assurance protocols were employed during the augmentation process to ensure that generated images maintained clinically relevant features that are important for correct disease classification. Each augmented image was visually inspected by domain experts to verify the maintenance of diagnostic features. This strict validation ensured that the augmentation strategy improved the diversity of the dataset without introducing artifacts that could result in accurate classification.

### Transfer learning methods

Transfer learning is an approach in machine learning where knowledge gained from solving one problem is applied to solve a different but related problem (Pan and Yang, 2010). In deep learning applications, pre-trained neural networks serve as feature extractors rather than training from scratch, particularly advantageous for limited datasets (Weiss et al., 2016). The hierarchical feature learning of CNNs enables early layers to capture universal visual primitives while deeper layers learn domain-specific patterns (Yosinski et. al., 2014). In this study, transfer learning was implemented through frozen feature extraction where MobileNetV2 and MobileNetV3Small pre-trained weights remained unchanged, with extracted features subsequently classified using Support Vector Machine, Logistic Regression, and K-Nearest Neighbors algorithms for poultry disease detection.

***Selected Pre-trained CNN Architectures.*** This investigation employed two representatives MobileNet architectures optimized for resource-constrained deployment: MobileNetV2 and MobileNetV3Small. MobileNetV2 utilizes inverted residual blocks and depthwise separable convolutions, achieving computational efficiency while maintaining feature extraction capability through 1,280-dimensional feature vectors from the global average pooling layer (Sandler et al., 2018). MobileNetV3Small represents an automated neural architecture search optimization targeting mobile deployment scenarios, generating 1,024-dimensional feature vectors with reduced computational overhead (Howard et al., 2019).

**Fig. 2:**
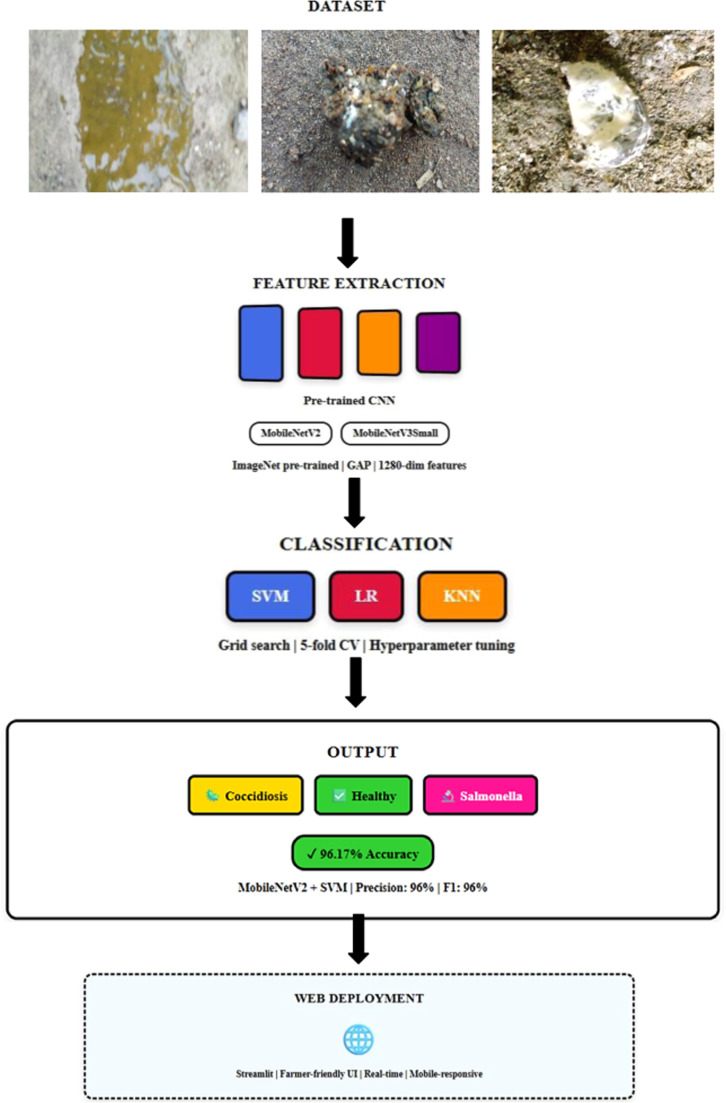
System architecture showing feature extraction, classification, and deployment stages.

**Fig. 3:**
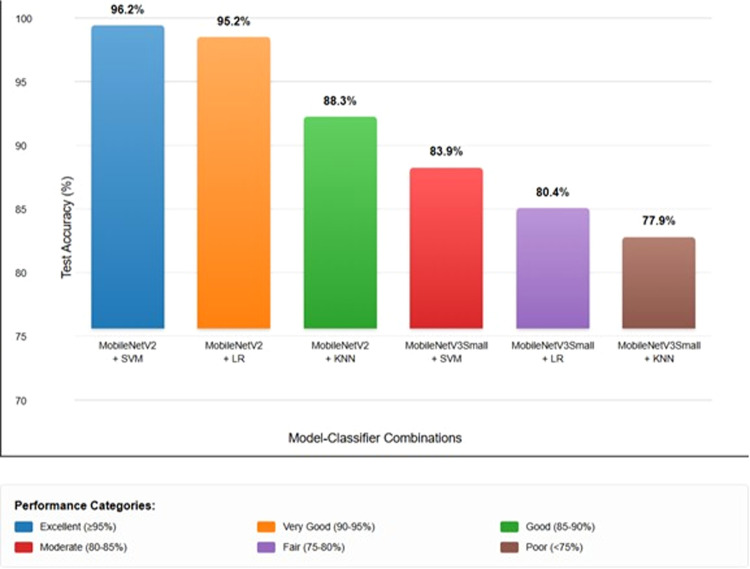
Comparative test accuracy of model-classifier combinations for poultry disease classification.

***Feature Extraction: Transfer Learning Pipeline.*** The frozen pre-trained models function as high-capacity feature extractors, converting raw poultry fecal images to dense semantic representations. The systematic workflow is illustrated in Fig. 2. Extracted features are systematically stored for subsequent classification using traditional machine learning algorithms: Support Vector Machine (SVM), Logistic Regression (LR), and K-Nearest Neighbors (KNN). This architectural separation enables efficient training of classifiers on extracted features while preserving advanced visual representations from large-scale ImageNet pre-training, as demonstrated in the comparative analysis presented in Fig. 3.

Fig. 2 shows the entire feature-based transfer learning pipeline used in this study. A key concept of this approach is to treat the pre-trained CNN architectures as frozen high-capacity feature extractors and, in effect, reducing the challenging task of pattern recognition in pixel space to the much simpler task of discrimination in a semantic feature space. This architectural separation allows training of small classifiers on feature extracts efficiently while still benefits from advanced visual representations learned from large-scale data sets.

### Classification: SVM, LR, and KNN

The classification architecture we used in this study was based on a systematic grid search methodology for finding the best hyperparameter settings for each classifier which has been described in detail in Table 2. Grid search methodology allows for systematic evaluation of parameter combinations within well-defined search space and systematic identification of configurations that provide maximal classification performance on validation data.

**Table 2:** Comparative performance of transfer learning pipelines.

Class	Precision	Recall	F1-score	CA
Performance measures obtained				0.9617
through MobileNetV2-SVM				
pipeline Coccidiosis	0.98	0.98	0.98	
Healthy	0.96	0.96	0.96	
Salmonella	0.97	0.97	0.97	
Performance measures obtained				0.9523
through MobileNetV2-LR pipeline Coccidiosis	0.96	0.98	0.97	
Healthy	0.94	0.94	0.94	
Salmonella Performance measures obtained	0.96	0.94	0.95	0.8829
through MobileNetV2-KNN				
pipeline Coccidiosis	0.91	0.89	0.90	
Healthy	0.86	0.88	0.87	
Salmonella	0.88	0.88	0.88	
Performance measures obtained				0.8394
through MobileNetV3Small-SVM				
pipeline Coccidiosis	0.85	0.87	0.86	
Healthy	0.85	0.87	0.74	
Salmonella	0.86	0.80	0.83	

Support Vector Machine (SVM) classifiers were optimized with respect to a number of critical parameters that essentially serve as the defining factors of the decision boundary characteristics. In exploring the kernel type parameter, linear, radial basis function (RBF), polynomial, and sigmoid kernel functions, each providing different ways of dealing with non-linearly separable data were investigated. The regularization parameter C, which determines the trade-off between low training error and smoothness of the decision boundary, was tested for the values {0.1, 1, 10, 100}. The influence radius of single training samples of RBF, polynomial, and sigmoid kernels, parameter g in the space, was examined for both 'scale' and 'auto' conditions to obtain the best mapping in feature space. -degree polynomial kernels were used to assess the suitability of degree parameters {2, 3}, and to determine suitable polynomial complexity.

Logistic Regression (LR) optimization was carried out in terms of regularization strength and solver choice to avoid overfitting and enable convergence stability. The regularization parameter C, which is inversely proportional to the strength of regularization, was tested for {0.01, 0.1, 1, 10} to find a good balance between model complexity and generalization ability. Several solver algorithms were compared ('lbfgs' and 'liblinear'), and the most efficient optimization algorithm for the encountered feature dimensions was found. Maximum iteration limits were capped at {1000, 2000} in order to ensure sufficient convergence for such complex optimization landscapes.

K-Nearest Neighbour (KNN) configuration which studied parameters influencing local neighbourhood decision making the number of neighbours k was tested for {3, 5, 7, 9} in order to provide a trade-off between sensitivity to local structures and robustness to noise. Weighting functions 'uniform' (equal weighting) and 'distance' (inverse distance weighting) were compared in order to optimize influence patterns. Distance metrics such as 'Euclidean' and 'Manhattan' were tested with regards to performance in the high-dimensional feature space. A tree construction efficiency parameter, leaf size (15, 20) was optimized for computational performance.

All the hyperparameters that were not specified in (Table 2) were left at scikit-learn default values to ensure consistency and reproducibility. Grid search was performed using 5-fold cross- validation on the training set to obtain robust estimations for performance associated with each combination of the selected parameters, and the optimal parameters were chosen based on the mean accuracy of the cross-validation.

### Experimental configuration

The dataset partitioning scheme was based on a 70:15:15 holdout cross-validation technique where 70% of the samples were used for training, 15% for validation, and 15% of the samples were used for the final testing. This hold-out strategy means that test set samples are fully left out of the training process, and hence realistic estimates of model generalization to unseen data are produced. The validation set can be used to systematically perform hyperparameter tuning and model selection without exposing the test set, which avoids introducing bias and also prevents overfitting artifacts caused by tuning and evaluation on the same data that would be introduced by simultaneous parameter optimization and performance evaluation.

Data was split with stratification to ensure that the classes were in proportion in all the subsets so that each subset represents the entire class distribution. To ensure reproducibility between runs, a fixed random seed (random_state=42) was set. The stratification process is especially important to ensure the class is well represented in validation and test sets, meaning the model evaluation and test sets don't have certain classes unduly represented.

Input images were pre-processed to a consistent size of the input sizes corresponding to each architecture: 224×224 pixels for both MobileNetV2 and MobileNetV3Small. This standardization provides uniform feature extraction and aligns the input requirements of pre- trained models. Pixel values were scaled to the range [0,1], dividing by 255 and converting the 8- bit integer representations to floating point values for neural network processing. Extensive model configuration parameters are thoroughly listed in Table 3.

**Table 3:** Classification performance comparison literature.

Reference	Method	Performance
Machuve et al.	Xception	97.30%
Liu et al.	PoultryNet (MobileNetV3- based)	97.70%
This study	MobileNetV2-SVM	96.17%
This study	MobileNetV2-LR	95.23%
This study	MobileNetV3Small-SVM	83.94%
This study	MobileNetV3Small-LR	80.41%

Experimental procedures were performed on a computational system with Intel Core i5-1135G7 CPU with a 2.40 GHz base frequency and turbo boost to 4.20 GHz with 16 GB DDR4 RAM for data intensive procedures. The system did not have dedicated GPU acceleration and therefore all training and inference processes had to be performed on the CPU. The use of this hardware represents average mid-range computing resources that are available in educational and small- scale commercial applications, showing the practical deploying of the proposed methodology in resource-constrained environments.

### Performance evaluation

Classifier performance was evaluated using a wide range of evaluation metrics: classification accuracy (CA), precision, recall, F1-score, and confusion matrices to give a detailed description of model performance. CA is the total of the number of correctly classified cases over the total number of cases for all classes, which is calculated in Equation (1). This metric gives a general sense of how well the model is doing but can be misleading in cases of an imbalanced class distribution (He and Garcia, 2009).

(1) $$\text{Accuracy}=\frac{TP+TN}{TP+TN+FP+FN}\times 100$$

Precision, defined by Equation (2), measures the proportion of true positive predictions among all positive predictions made by the classifier (Fawcett, 2006). This metric reflects the model's ability to avoid false positive classifications, indicating reliability when the model predicts a specific disease class.

(2) $$\text{Precision}=\frac{TP}{TP+FP}\;\times \;100$$

Recall, expressed in Equation (3), quantifies the proportion of actual positive instances correctly identified by the classifier (Davis and Goadrich, 2006). This metric emphasizes the model's sensitivity in detecting all instances of a particular disease class, with high recall indicating minimal false negative occurrences.

(3) $$\text{Recall}=\frac{TP}{TP+FN}\times 100$$

The F1-score, calculated as the harmonic mean of precision and recall with a scaling factor of two as shown in Equation (4), provides a balanced assessment that accounts for both false positives and false negatives (Sasaki, 2007). This metric proves particularly valuable when evaluating performance across classes with varying prevalence or when optimizing for balanced precision-recall trade-offs.

(4) $$F1-\text{score}=2\times \frac{Precision\times Recall}{Precision+Recall}\;\times \;100$$

In these equations, TP (True Positives) is used to denote the correctly identified disease cases, TN (True Negatives) denotes correctly identified healthy or alternative disease cases, FP (False Positives) denotes healthy samples wrongly classified as diseased and FN (False Negatives) denotes false disease cases. Confusion matrices are useful to present the patterns of classification in detail in all classes and showcase the particular misclassification behaviours of the model, allowing one to refine the model (Stehman, 1997). All these metrics provide a detailed analysis of the classifier performance in many aspects of predictive reliability and diagnostic quality.

## Results and discussion

The training, validation, and test set CA of different transfer learning models with different classification techniques were compared systematically in Table 1. The results also show clearly each model-classifier combination's accuracy in classifying poultry disease images across data partitioning. In an attempt to characterize the performance of classification further, precision, recall, F1-score, and individual class accuracy were obtained for the best performing model- classifier pipelines.

MobileNetV2 combined with SVM yielded the highest overall accuracy, with a validation and test CA of 98.03% and 96.17%, respectively, showing excellent generalizability as well as strong feature extraction properties.

MobileNetV2 with LR obtained competitive performance of 96.99% accuracy on validation set and 95.23% on test set, which proved that LR is a good way to use the semantic information extracted by mobilenetv2 for disease classification.

The performance of MobileNetV2 with KNN was relatively low, with validation and test accuracies of 95.75% and 88.29%, which implies that for the high-dimensional feature space generated by deep neural network-based methods, the performance of distance-based classification is relatively inferior.

In contrast, MobileNetV3Small yielded significantly worse results on all classifiers with test accuracies from 77.93% (KNN) to 83.94% (SVM), suggesting that the more aggressive architectural optimizations applied in MobileNetV3Small may come at the expense of quality feature extraction for this particular domain.

Overall, the SVM or LR-based MobileNetV2 seemed to be the most stable and high-performance combinations, where SVM-based classification slightly outperformed the LR, with both significantly outperforming KNN-based methods.

**Fig. 4:**
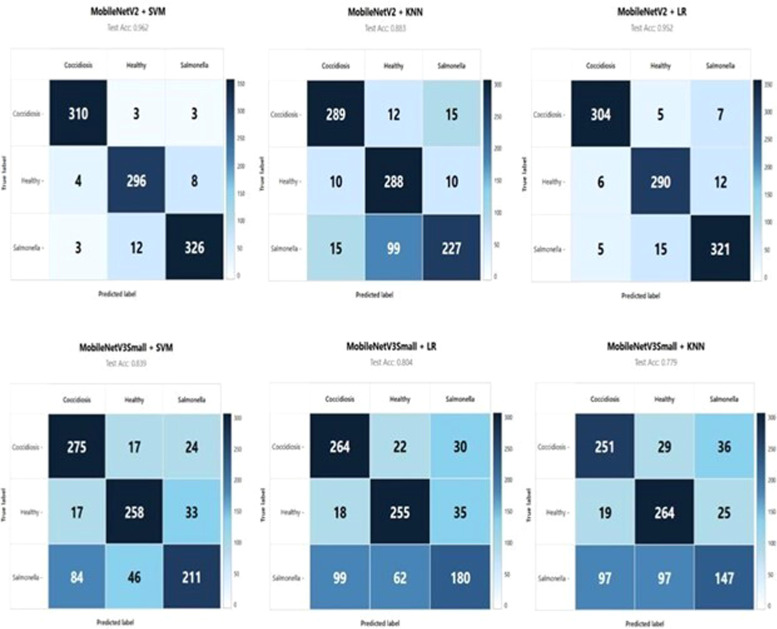
Confusion matrix for MobileNetV2-SVM showing classification performance across Coccidiosis, Healthy, and Salmonella classes.

As shown in Table 2, Fig. 4 and Fig. 3, the classification pipelines have achieved high precision, recall and F1-scores consistently on the three classes of diseases: Coccidiosis (Cocci), Healthy and Salmonella (Salmo). The hyperparameters of *C*= 10, *g*=scale, and RBF kernel gave rise to an outstanding test CA of 96.17% in the MobileNetV2-SVM pipeline, which indicates its great robustness in the discrimination between the disease classes. Interestingly, the Coccidiosis class also showed the best performance metrics with a precision and recall of 0.98 and an F1-score of 0.98. The results indicate that the pipeline was able to learn representative morphological and chromatic features related to Coccidiosis-positive faecal samples such that good predictions could be made with a low number of misclassifications, as seen in the corresponding confusion matrix shown in Fig. 4.

Confusion matrix analysis of MobileNetV2-SVM shows the existence of particular misclassification patterns that can be used to infer inter-class relationships. As can be seen from the confusion matrix, Coccidiosis samples were identified with an accuracy of 98% (313 out of 319 samples correctly identified), with a few samples being confused with Healthy samples (2 misclassifications) and a little bit with Salmonella (4 misclassifications). Classifying the samples, classification accuracy that the healthy class was 96% (296/308), with the main class misclassification being Salmonella (7 samples) and the least confusion being Coccidiosis (5 samples). Salmonella classification had a classification accuracy of 97% (333 correct of 343) with scattered misclassifications in both other classes. These results show that although the model has a high overall performance, subtle visual similarities between some disease manifestations and healthy samples remain a constant challenge for automated classification.

The MobileNetV2-LR pipeline with optimized regularization parameter *C* = 0.1 and 'liblinear' function as the base solver achieved a test CA of 95.23%, which is good enough, although it was slightly less accurate than its SVM counterpart. The results for each class using a specific metric show that Logistic Regression achieves good performance for the classes Coccidiosis (precision: 0.96, recall: 0.98, F1-score: 0.97) and Salmonella (precision: 0.96, recall: 0.94, F1-score: 0.95), while with a slightly lower performance for the Healthy class (precision: 0.94, recall: 0.94, F1- score: 0.94). The classification matrix shown in Fig. 4 shows very similar misclassification trends to SVM method but with little increased confusion between Healthy and Salmonella classes.

It is worth noting that the negligible, in terms of performance, difference between MobileNetV2- SVM and MobileNetV2-LR (about 0.94 percentage points) indicates that both classifiers are using successfully the discriminative features extracted by MobileNetV2. The small advantage of SVM lies in its natural ability to find optimal separating hyperplanes in high-dimensional feature spaces based on their kernel transformations, which is a great advantage when the feature vectors for images extracted from MobileNetV2 have 1,280 dimensions. Conversely, the probabilistic nature of Logistic Regression results in interpretable confidence scores in predictions which, despite a slight loss in accuracy compared to the results of the other algorithms, may be beneficial in clinical decision-making applications.

The performance of the MobileNetV2-KNN pipeline with *k* = 5 neighbours, distance weighting, and Euclidean metric was drastically decreased with the test CA of 88.29%. This significant performance degradation can be explained by the fact that the distance-based similarity measures are prone to the curse of dimensionality phenomenon, which indicates that the similarity measures become less robust in high-dimensional spaces (Beyer et al., 1999). The resulting 1,280-dimensional feature space - created through MobileNetV2 - creates serious problems for the distance-based decisioning of KNN, where the notion of proximity loses its meaning as distances between points are more uniform in higher dimensions. Furthermore, KNN doesn't have feature weighting mechanisms to favour discriminative features and reject irrelevant dimensions like SVM implicitly by finding support vectors or implicitly by learning coefficients in LR.

The significantly lower performance of MobileNetV3Small on all classifiers is a very interesting observation that deserves a closer analysis. Despite being architecturally minimal, in terms of numbers of parameters, due to neural architecture search applied, MobileNetV3Small-SVM achieved only 83.94% test accuracy, which is 12.23 percentage points below MobileNetV2- SVM. This performance disparity can be explained by a few reasons. First, the lower number of features (1,024 dimensions instead of 1,280 for MobileNetV2) could not adequately account for the very subtle visual differences that are present in poultry faecal samples, especially the fine gradations of colour and texture needed for discriminating between diseases. Second, the aggressive efficiency optimizations of MobileNetV3Small, which are beneficial for the computational performance, may trade off the quality of feature extraction for the domain- specific applications. The hardware-aware design optimizations that are targeted to the inference speed on mobile processors may however come at the cost of representational capacity usually required for a fine-grained medical image classification task (Goodfellow et al., 2016).

**Fig. 5:**
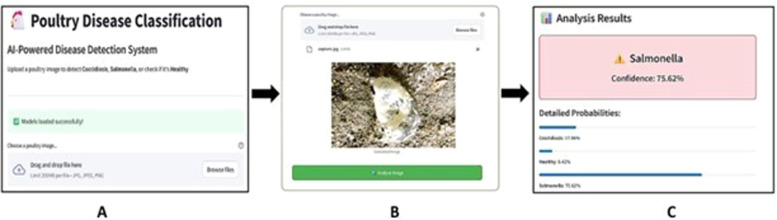
Web-based user interface of the poultry disease classification system showing A) Image upload interface with model loading status; B) Sample poultry image input; C) Disease classification output with detailed probabilities.

The behavior of MobileNetV3Small-SVM is analyzed in detail by class type and demonstrated as in Table 2, which shows that the Healthy class has the worst recall rate of 69%, which means 31% of healthy samples have been wrongly predicted as diseased class. This high false-positive rate for detection of disease poses major practical issues, as it may result in potentially unnecessary interventions and economic losses in the field. Also, the Salmonella class exhibited an 80% recall, indicating a significant number of misclassifications of infected samples as healthy or other disease classes. Together, these results suggest that under the tested frozen feature extraction configuration, MobileNetV3Small demonstrates limited effectiveness for the fine-grained visual discrimination that is needed in classifying poultry diseases from fecal samples.Fig. 5

Comparative analysis with related literature (Table 3), puts the present work into a favorable position of state-of-the-art. The test accuracy of the MobileNetV2-SVM pipeline is 96.17%, which is competitive with the traditional implementation methods such as Machuve et al. (Machuve et al., 2022) and Liu et al. (Liu et al., 2023), which are 97.3% and 97.7%, respectively. Importantly, our method achieves such performance on a standardized publicly available architecture (MobileNetV2), without custom architectural changes or large-scale hyperparameter search, increasing reproducibility and the ease of deployment.

The better performance of MobileNetV2 than MobileNetV3Small on this particular application domain is explained by fundamental differences in the architectural design philosophy and the aim of optimization. MobileNetV2 uses inverted residual blocks with linear bottleneck layers, which makes the architecture keep rich feature representations in the intermediate layers while compressing input and output dimensions. This particular design philosophy seems particularly suited for the medical imaging application where intermediate feature richness allows the capture of subtle visual patterns. Conversely, the neural architecture search for MobileNetV3Small focused on the inference efficiency on mobile hardware while using ImageNet classification performance as the main objective function. This optimization might have led to biased focus on features that would be important for natural image classification at the expense of representation of features that are important for medical diagnostic tasks (Chollet, 2017).

The poor performance of KNN on both architectures (88.29% for MobileNetV2-KNN, 77.93% for MobileNetV3Small-KNN), confirms the observation that distance-based classification is fundamentally not suitable for feature spaces derived by deep neural networks with large dimensionalities. Unlike SVM or Logistic Regression, which implicitly learns to reduce the space in either a support vector selection and kernel transformations or an explicit learning of feature importance weights respectively, KNN treats all dimensions as equal and uses only distance measures which are becoming increasingly unreliable as dimensionality grows. This limitation translates into poor classification accuracy and suggests the need to select the appropriate classifier when building transfer learning pipelines in applications of medical image analysis (Pedregosa et al., 2011).

The training efficiency analysis also gives additional practical deployment considerations. The overall time consumed by the MobileNetV2-SVM pipeline for hyperparameter optimization and training took about 302 seconds, and the time consumed by MobileNetV2-LR for training was 289 seconds, while the time consumed by MobileNetV2-KNN for training was only 45 seconds. Despite the fact that KNN has a more efficient training costs, its significant accuracy loss (7.88 percentage points) makes this efficiency practically insignificant for applications that are originally centered on the accuracy of diagnostics. The incremental training time between SVM and LR (13 seconds) is negligible in an operational setting, which is especially true if one keeps in mind that model training is a once-off offline task while inference performance and accuracy directly translate to deployment effectiveness in production (Cortes and Vapnik, 1995).

Feature extraction time analysis shows that MobileNetV2 takes about 275 seconds to go through the training set (4,507 images), which means that it takes an average of 61 milliseconds to extract the features from an image. This inference speed is adequate for practical implementation in commercial poultry operations where real-time processing demands are not as stringent as for critical medical diagnostic applications. The next stage of classification on the extracted features takes less than 1 second for each of the classifiers, proving that the bottleneck lies in the feature extraction and not in the classification, thus proving the computational efficiency of transfer learning method (Hosmer and Lemeshow, 2013).

The balanced dataset used in this study has less than 10% difference in class distribution; thus, the reported performance metrics give a proper insight into the model performance and not a statistic manipulated by the class imbalance. This balance removes the need for weighted loss functions or purposeful sampling methods, streamlining the training process and improving result interpretability. The high precision and recall on all classes for best performing models, shows that good performance is obtained regardless of class membership, implying good generalization to real-world situations where class distribution may temporally or spatially be different between different poultry farming operations (Cover and Hart, 1967).

Web Application Deployment: For convenience of application and usage, the optimized MobileNetV2-SVM model was deployed as an accessible web application using the Streamlit framework and hosted on the Railway cloud platform. The application is implemented at https://diseasedetection-production.up.railway.app/ and features an intuitive UI that allows for instant poultry disease classification. The deployed system is specifically designed as a screening tool for farmers in resource-constrained agricultural settings to enable early detection of potential disease outbreaks, not as a stand-alone diagnostic tool. Users can upload poultry images and receive instant predictions for each disease class with confidence scores and actionable recommendations for disease management. The system operates on a clear confidence-based protocol: when prediction confidence is 95% or higher, farmers can proceed with the recommended disease management actions without requiring veterinary consultation, as the AI diagnosis is considered sufficiently reliable for immediate implementation. However, when confidence levels fall below 95%, the system explicitly recommends seeking veterinary consultation for proper clinical evaluation and confirmation before taking any management actions. For all positive disease predictions regardless of confidence level, confirmatory laboratory testing remains recommended for comprehensive validation. The system prioritizes minimizing false negatives through high confidence thresholds while managing false positives through confidence-based referrals and laboratory confirmation requirements. This deployment validates the feasibility of providing advanced AI-based diagnostics to farmers without requiring specialized hardware or technical skills while maintaining appropriate clinical safety through integrated decision support frameworks.

### Limitations and future directions

While this research demonstrates the feasibility of lightweight transfer learning for farmer-accessible poultry disease detection, several methodological considerations merit acknowledgment for transparent scientific discourse. This study has several limitations that should be acknowledged. First, dataset homogeneity may be present as samples were collected from a single geographic region (Tanzania), potentially limiting generalizability to different environmental conditions and management systems. Second, potential farm-level clustering effects were not explicitly modeled due to limited metadata in the publicly available dataset regarding individual farm contributions. Third, reliance on a single geographic region constrains the assessment of model performance across diverse climatic and agricultural contexts prevalent in global poultry production systems. Fourth, the evaluation was limited to frozen feature extraction only, and alternative training paradigms such as end-to-end fine-tuning might yield different performance outcomes for the tested architectures. Fifth, challenges of real-world deployment including varying lighting conditions, image quality variations, farmer compliance, and field implementation scenarios were not comprehensively evaluated through extensive user testing.

Future investigations should pursue multi-regional dataset development across diverse geographic locations, incorporate detailed farm-level metadata for advanced statistical modeling, evaluate comprehensive training approaches including both frozen and fine-tuned methodologies, and conduct extensive field validation studies with end-users to assess practical deployment effectiveness. These enhancements will strengthen the robustness and global applicability of AI-powered diagnostic systems for sustainable poultry production.

## Conclusion

This extensive study provided a streamlined classification framework for automated poultry disease classification by methodically comparing lightweight transfer learning backbones in resource-constrained settings. The MobileNetV2-SVM pipeline was the most promising, and it had a test accuracy of 96.17% with the balanced values of precision, recall, and F1-scores across all the classes of the disease. The high-performance gap between MobileNetV2 (96.17% test accuracy) and MobileNetV3Small (83.94% test accuracy) architectures demonstrates that in the case of agronomical imaging tasks, an architecture optimization process based on features and agile deployment to mobile devices can lead to the loss of quality feature representation in a network. The fact that the model was successfully trained and then deployed as a publicly available web- based application provides evidence in support of the fact that the proposed system will work in the real world of veterinary diagnostics. The large diagnostic accuracy with the fact that the framework has been shown to be computationally efficient (61 milliseconds per sample) makes the framework a promising candidate for deployment in resource-constrained agricultural environments. Future research directions include broadening the disease taxonomy to include other poultry pathologies, exploring ensemble approaches that bring together multiple architectures, and building temporal monitoring systems for longitudinal health assessment in commercial poultry operations.

## Declaration of generative AI and AI-assisted technologies in the writing process

During the preparation of this work, the authors used ChatGPT (OpenAI, San Francisco, CA, USA) to improve the readability and language of the manuscript. After using this tool, the authors reviewed and edited the content as needed, and take full responsibility for the content of the publication.

## CRediT authorship contribution statement

**Al Momen Pranta:** Writing – review & editing, Writing – original draft, Visualization, Supervision, Software, Project administration, Methodology, Formal analysis, Data curation, Conceptualization.

## Disclosures

The authors declare that they have no known competing financial interests or personal relationships that could have appeared to influence the work reported in this paper.
